# The prevalence of prolonged grief disorder (PGD) after the natural disasters: A systematic review and meta-analysis

**DOI:** 10.1016/j.puhip.2024.100508

**Published:** 2024-05-16

**Authors:** Armin Zareiyan, Ali Sahebi, Bayram Nejati-Zarnaqi, Reza Mosaed, Rahman Berdi Ozouni-Davaji

**Affiliations:** a Public Health Department, Nursing Faculty, Aja University of Medical Sciences, Tehran, Iran; b Department of Medical Emergencies and Health in Disasters and Emergencies, Ilam University of Medical Sciences, Iran; c Non-Communicable Diseases Research Center, Ilam University of Medical Sciences, Ilam, Iran; d Department of Health Management and Economics, Faculty of Medicine, Aja University of Medical Sciences, Tehran, Iran; e Infectious Disease Research Center, Aja University of Medical Sciences, Tehran, Iran; f Student Research Committee, Aja University of Medical Sciences, Tehran, Iran; g Health Management and Social Development Research Center, Golestan University of Medical Sciences, Gorgan, Iran

**Keywords:** Grief, Prolonged grief disorder, Natural disaster, Meta-analysis, Disaster

## Abstract

**Objective:**

The failure to detect PDG and lack of providing essential interventions accordingly can disrupt the lives of survivors of natural disasters years after the death of their loved ones. The present study aims to investigate PGD after natural disasters using a systematic review and meta-analysis.

**Study design:**

This study was performed according to the Preferred Reporting Items for Systematic Review and Meta-Analysis guidelines.

**Methods:**

With the focus on the prevalence of PGD after natural disasters, studies conducted until the end of 2021 were collected without a time limit. To do this, reputable databases such as PubMed, Web of Science, Scopus, Embase, Google Scholar, and Science Direct were used. The random effects model was used to perform a meta-analysis of the studies. To check the heterogeneity between the studies, the I2 index was used. The publication bias of the study was evaluated using Begg's test. Data were analyzed using the STATA software.

**Results:**

Primarily, 2566 studies were collected based on the initial search, from which 12 final studies were entered into the analysis. The results showed that the prevalence of PGD after natural disasters was 38.81 % (95 % CI: 24.12–53.50, I2 = 99.7 %, p = 0 < 001).

**Conclusions:**

It is recommended that policies and plannings of the organizations responsible for disaster management be prepared to send specialized teams of psycho-spiritual counseling, quickly accommodate the injured, and reconstruct the damaged buildings in the shortest time possible.

## 1 Introduction

Victims of natural disasters face negative and harmful impacts like the death of loved ones, loss of property, monetary issues, and physical and emotional wounds that are expected a very long time to recuperate [1]. The effects of natural disasters can be classified as (i) direct loss such as physical damages or deaths; (ii) indirect loss such as loss of income; and (iii) intangible effects of mental injuries caused by direct/indirect losses [2]. Bereavement is one of the most painful experiences in humans' lives. It may happen following the death of a loved one by any reason such as natural disasters and can lead to physical, psychological, spiritual, and social consequences [3]. As it is required to take medical measures in physiological disorders in order to restore the previous balance of the body, prolonged grief also requires counseling and treatment measures [4].

Grief after the death of a loved one is an essential and natural process that many people experience. Most symptoms of grief, such as anger, sadness, and a sense of guilt, decrease after 6 months [5]. However, some people lack the essential skills to overcome grief. As a result, other more complicated problems emerge including Prolonged Grief Disorder (PGD) [4]. Among the factors disrupting the natural grief process is the suddenness and unexpectedness of the death of a loved one. This, as a direct consequence of natural disasters, would lead the bereaved to suffer from prolonged grief [6]. Kristensen et al. (2010) [7] showed that 47.7 % of the people studied were directly and indirectly affected by the 2004 tsunami in Southeast Asia and were detected to have PGD. The death of a child or spouse, and the lengthening of the time to identify the body were among the factors causing PGD [7].

Lately, PGD has been recognized as a new diagnosis related to the adjustment disorder. It has recently been included in the Diagnostic and Statistical Manual of Mental Disorders, Fifth Edition (DSM-V), as Persistent Complex Bereavement Disorder (PCBD) [8]. Also, it has been mentioned in the latest edition of the International Classification of Diseases (ICD-11) as a diagnosis [9]. The difference between PGD and PCBD is only semantic and the level of agreement between the original PGD test and the PCBD test is high [10].

Studies show differences between natural grief and prolonged or complex grief [11]. PGD is present when, after the death of someone close at least 12 months ago, one still experiences long-lasting symptoms such as prolonged yearning for the deceased, intense sadness, thinking a lot about the deceased, difficulty in accepting death, difficulty in recalling positive memories of the deceased, anger at loss, and excessive avoidance of remembering death of the deceased [8]. Ghaffarinejad (2007) showed that 43–76 % of the cases studied suffered from PGD after the earthquake in Bam [12]. In his study on the survivors of the Sichuan earthquake in China, Li (2015) showed that 71.1 % of the population under study were suffering from complex grief disorder. Close relationship with the deceased, the presence of Post-Traumatic Stress Disorder (PTSD) symptoms, physical injury, and scary experiences in earthquakes were among factors causing complex grief disorder [13].

Inattention to the treatment of PGD will increase the risk of negative health consequences such as impairment in the spiritual health of the survivors [14], depression, increased alcohol consumption, suicide, physical disorders, and reduced social participation [15]. Conducting research and sufficient prior knowledge about the pattern of diseases and injuries caused by disasters are key factors in better planning and reducing the impact of disasters on the affected population [16].

Literature reviews showed that no comprehensive review study had been conducted so far to investigate the prevalence of prolonged grief disorder after the occurrence of natural disasters. Thus, the main objective of this research is to study the prevalence of prolonged grief disorder after natural disasters using a systematic and meta-analysis review. The results of this research can be used by health care policymakers for raising awareness and enabling health service providers to reduce the harmful consequences of natural disasters in an affected society.

## 2 Methods

This review was conducted using the PRISMA (Preferred Reporting Items for Systematic reviews and Meta-analyses) guideline [17]. The protocol of this review study was registered in the PROSPERO (International Prospective Register of Systematic Review) with code CRD42022304161.

**Search strategy:** Information resources in English and Persian were used including PubMed, Web of Science, Scopus, Embase, Google Scholar, Magiran, SID, and Science Direct. Also, related studies were found by searching for valid English keywords with equivalent Persian keywords. Keywords, search fields, and operators were used to develop a search strategy in databases. Searches were conducted in both Persian and English without time limits until the end of 2022. The search strategy in various databases is mentioned in Table 1.

**Table 1:** The characteristics of studies chosen for meta-analysis.

First Author	Year of Study	Location	Assessment Instrument	Type of Disaster	Sample Size	Male	Female	Mean Age (SD)	Follow up (months)	Prevalence%
Yi [31]	2017	China	PG-13	Earthquake	1464	604	860	–	84	8.47
Xu [53]	2014	Australia	ICG	Earthquake	226	0	226	39.8	34	88.9
Tsutsui [54]	2014	Japan	ICG	Earthquake	82	15	67	45.8	8	9.8
Shear [3]	2011	USA	ICG	Hurricane	3088	–	–	–	19	15.3
Rajkumar [30]	2015	India	DSM-5	Tsunami	643	318	325	33.7	9	14.2
Li [13]	2015	China	ICG	Earthquake	803	297	506	46.7	12	71.1
Kristensen [7]	2009	Norway	ICG	Tsunami	130	63	67	45.7	26	47.7
Johannesson [41]	2011	Sweden	ICG	Tsunami	486	203	283	–	14	43.5
Hu [38]	2015	China	ICG	Earthquake	271	123	148	44.9	19	79
Ghafari [12]	2007	Iran	ICG	Earthquake	400	211	189	37.8 ± 12	34	76
Ergun [40]	2021	Turkey	PG-13	Earthquake	495	229	266	36.44	96	8.9
Harms [55]	2015	Australia	ICG	Bushfire	294	117	177	52.49	46	3.5

**Inclusion criteria:** All the studies conducted regarding the prevalence of abnormal grief after natural disasters were used in this study. This includes PGD, Complicated Grief (CG), Prolonged Grief (PG), Persistent Complex Grief Disorder (PCGD), Pathological Grief (PG), and PCBD.

**Exclusion criteria:** Exclusion criteria included review studies, intervention studies, and letters to the editor. Also, studies that reported only the mean of PGD were not included.

**Study selection:** At first, all the studies found initially were entered into the Endnote 7 software to manage the resources. Then, the title and abstract of 2140 studies were screened after removing duplicates. In the next step, two researchers independently studied 131 possible related studies in detail and finally selected 12 studies as the ultimate collection. Every disagreement between the researchers was resolved by a third party.

## 3 Quality assessment and data extraction

### 3.1 Two tool-independent researchers

The Appraisal Tool for Cross-Sectional Studies (AXIS tool) [18] was used to evaluate the quality of the selected studies. The tool has a 0–20 score range. Studies with scores higher than 12 were selected for meta-analysis. All the final articles included in the study process were reviewed by two researchers independently using a previously prepared checklist to gather data. The checklist also included the first author, research year, type of natural disaster, follow-up time, average age, the tools used, sample size, the number of men and women, and the prevalence of PGD. Any disagreement between the researchers was resolved by a third party.

### 3.2 Statistical analysis

A simple random effects model was used for meta-analysis. The heterogeneity between studies was calculated using the I2 index. The heterogeneity less than 25 %, within 25–50 %, within 50–75 %, and above 75 % respectively shows no heterogeneity, medium, high, and very high heterogeneity [19]. The subgroup analysis was used to identify the source of heterogeneity. Publication bias in the study was checked using the Begg test. Meta-regression was used to investigate the relationship between the year of the study, the follow-up time and the prevalence of PGD. The data was analyzed using the STATA software (ver. 14)

## 4 Results

**Fig. 1:**
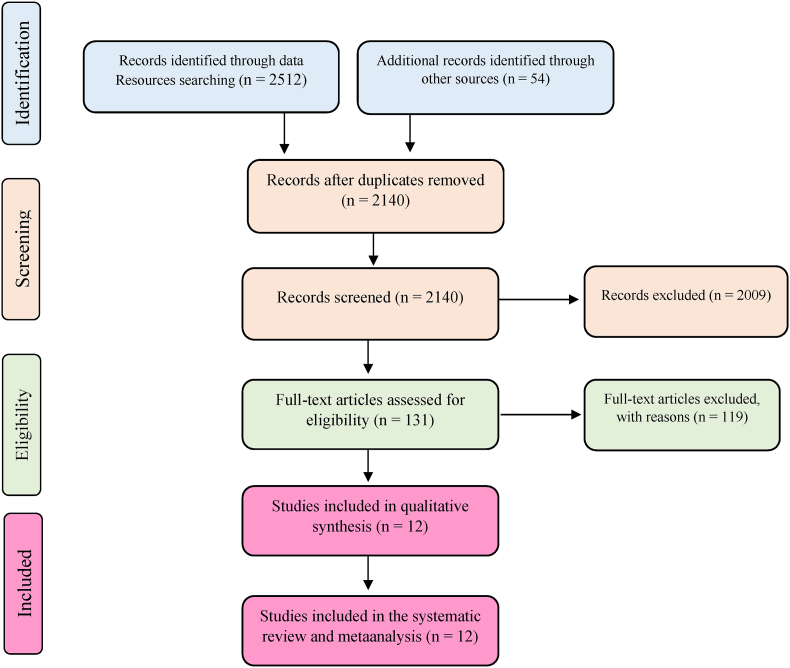
Flowchart of the selection of studies based on PRISMA.

**Fig. 2:**
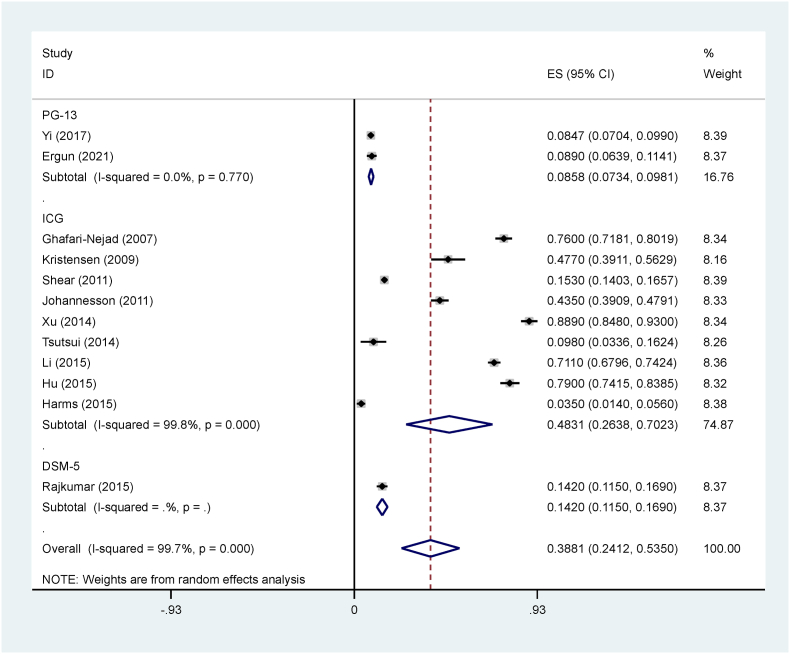
PGD rate and confidence interval of 95 % for each of the examined studies and all studies. The point in the middle of each line segment indicates the rate of PGD, and the length of the line segment represents confidence interval of 95 %.

In this review study, 2566 articles were identified through an initial search, and 2330 studies were screened after removing duplicates. Among them, 12 studies were selected and their quality was evaluated. Ultimately, all the remaining 12 studies entered the meta-analysis stage (Fig. 1). The methodology of all the selected studies was cross-sectional (Table 1). In this study, the rate of PGD after natural disasters was reported as 38.81 % (95 % CI: 53.50-24.12, I2 = 99.7 %, p = 0 < 001). The I2 index showed a very high heterogeneity between the studies (Fig. 2).

### 4.1 Subgroup analysis, meta-regression, and publication bias

**Fig. 3:**
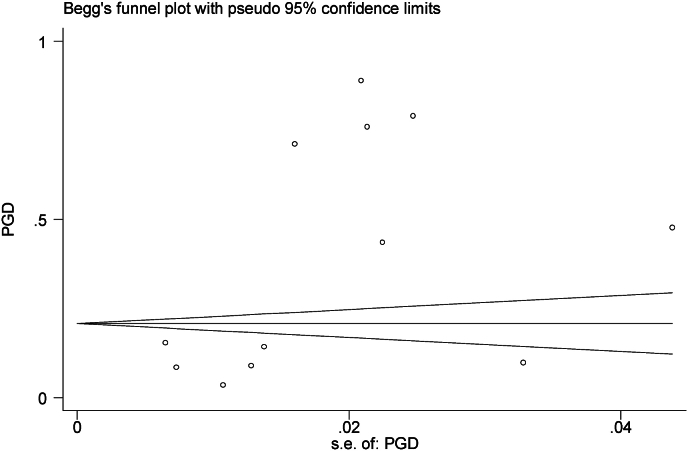
Publication bias based on Begg test.

**Fig. 4:**
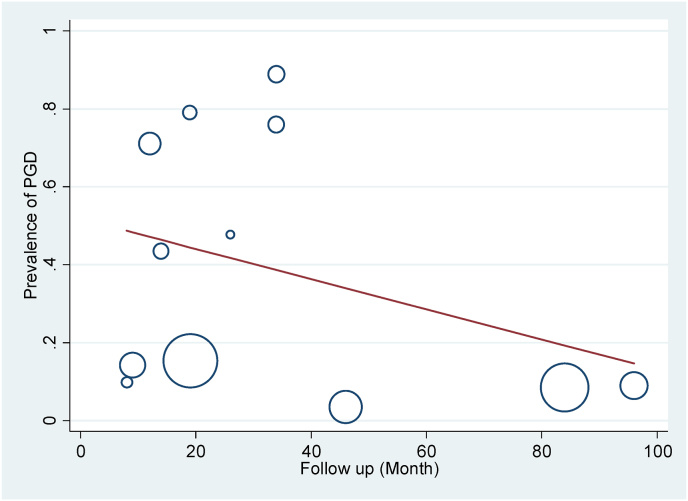
Meta-regression of PGD rate based on follow-up time.

**Fig. 5:**
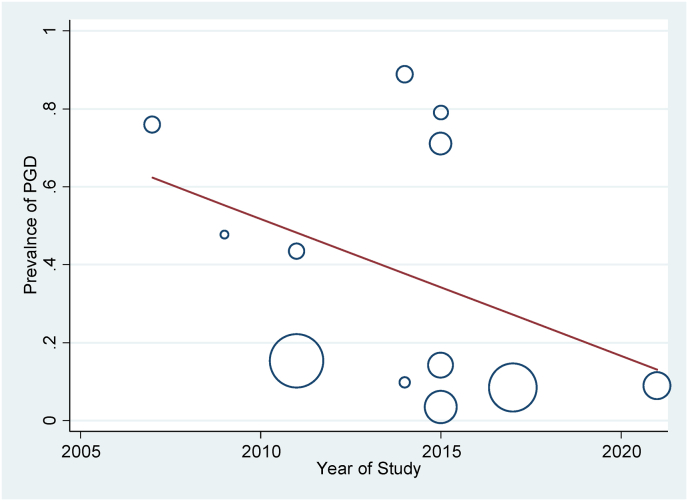
Meta-regression of PGD rate based on year of study.

In this study, subgroup analysis was used based on the type of tool used to measure PGD in the studies. The prevalence of PGD based on PG-13, ICG, and DSM-5 tools was respectively reported as 8.58 % (95 % CI: 7.34–9.81, I2 = 0.0 %, p = 0.770), 48.31 % (95 % CI: 26.38–70.23, I2 = 99.8 %, p = 0 < 001), 14.20 % (95 % CI: 11.50–16.90) (Fig. 2). Based on the subgroup analysis, the PGD rate in the PG-13 tool was without heterogeneity. The meta-regression results showed that the prevalence of PGD had a deceasing trend based on the research year and follow-up time (Fig. 3, Fig. 4). Based on the results of the Egger test, the publication bias (p = 0.075) was not significant in this research (Fig. 5).

## 5 Discussion

In the present study, the prevalence of prolonged grief disorder after natural disasters was investigated and 12 studies were meta-analyzed. The results of the research showed that the overall rate of PGD after natural disasters was 38.81 %. Studies about the prevalence of prolonged grief in victims after the-2004 tsunami in Southeast Asia showed that at least 50 % of the victims were diagnosed with PGD after 2 years of follow-up [7]. Research on the prevalence of PGD after the 9/11 attacks showed that 43 % of the victims had PGD after 3.5 years [20]. These statistics show that, compared with natural deaths, the prevalence of PGD after natural disasters and man-made disasters is high due to their difference in bereavement. An effective factor in the occurrence of PGD after natural disasters is the nature of death i.e., its suddenness, unexpectedness, and severity of injuries [21]. Other effective factors in the prevalence of PGD are as follows: simultaneous loss of several family members or other close relatives/friends [22], waiting to confirm the death of loved ones [23], and weak social support after natural disasters [24]. The prevalence of PGD in victims of COVID-19 was reported as 37.8 %. The factors of high prevalence of PGD were reported the unexpected death of Corona patients and the impossibility of holding funerals [25,26]. Therefore, in the crisis management phase, it is necessary to plan for reducing the prevalence of PGD after natural disasters. To do so, it seems vital to plan for the timely collection and management of corpses from the identification stage to the delivery of the dead bodies and the mourning ceremonies.

Religious and spiritual beliefs are key factors for the adaptation and resilience of people affected by natural disasters [27]. In addition, the cultural differences of the affected society and the religious and spiritual beliefs of the victims are mentioned as the effective factors in the occurrence of PGD in most studies [[28], [29], [30], [31]]. Recent studies show that the traumatic death of loved ones such as deaths after natural disasters including earthquakes causes spiritual crises, which in turn leads to the occurrence of PGD [32]. Unexpected deaths after natural disasters question the underlying beliefs of the bereaved about God, the world, and the purpose of being. The lack of proper response and the escalation of such doubts challenge the spiritual adaptation of the bereaved and divert the natural grief towards PGD [4]. It is suggested to provide spiritual rehabilitation programs for the bereaved and clinical pastoral care for those injured and hospitalized due to natural disasters.

Some research studies found that getting help from spiritual beliefs was one of the adaptation ways for the bereaved people. Communicating with God through prayers, finding a meaning for the death of the loved ones, and having a divine worldview can all reduce the symptoms of grief [33,34]. Bereaved people who profess stronger spiritual beliefs seem to resolve their grief more rapidly and completely after the death of loved ones than do people with no spiritual beliefs [35]. Spiritual rehabilitation of victims of natural disasters through spiritual counseling can improve their health and facilitate the grief process [36]. Spiritual rehabilitation through meaning-making and communicating with a supreme power (God) strengthens spiritual beliefs and the power of adaptation [37].

The meta-regression results of the present study showed that the prevalence of prolonged grief disorder after natural disasters had a decreasing trend based on the research year and follow-up time. Li et al. (2015) showed in his study that the prevalence of PGD after the earthquake was 71.1 % after 12 months [38]. The result was consistent with the decreasing trend over time found in a later study by Ergun (2021), which declared the prevalence of PGD to be 8.9 % at 96 months after the earthquake [30,38]. The results of research about the victims of the Wenchuan earthquake in China showed that the rate of PGD decreased from 79 % (1–1.5 years after the earthquake) [13,38] to 8.47 % after 7 years following the earthquake [31]. Evidently, time is an effective variable on PGD: over time the bereaved gets better along with the demise of a friend or a family member and the side effects of PGD gradually decline [39]. However, not providing serious consideration to the affected people after natural disasters and lack of preventive measures can bring about serious consequences such as committing suicide.

Among the collected 12 studies, 6 studies reported the amount of PGD based on gender [7,12,31,38,40,41]. In all these studies, the prevalence of PGD was higher in women. The Odds Ratio (OR) of PGD in women were 4.73 (95 % CI: 1.48–15.14), 2.35 (95 % CI: 1.13–4.90), and 1.87 (95 % CI: 1.26–2.77) respectively in the studies of Kristensen, Ergun, and Bergh [7,40]. After natural disasters, women are more exposed to gender-based violence [[42], [43], [44]]. Consequently, mental disorders are more prevalent among women [45,46]. Women are less involved in group work and social participation after natural disasters [47,48], resulting in their deprivation of the support of other disaster victims. The lack of a supportive social network can lead to the development of complicated grief reactions [4].

The closeness of the relationship between the bereaved and the deceased, being e.g., the spouse, child or a family member, is an important factor in the occurrence and continuation of PGD [49].

The prevalence rate of PGD among the people affected by the natural disasters depends on socio-economic support [50,51] available in the affected society after the disaster, acceleration in the construction of permanent residence [12], and psychological services provided to the survivors [52]. Such measures can have a decreasing trend in the treatment of PGD [52]. It is suggested that the organizations responsible for disaster management minimize the time taken for the families to return to their normal life by properly planning prior to any disaster. These measures should appropriately match the cultural, social, and economic context of the affected society.

A limitation of this study was the high heterogeneity of the investigations because of the number of various samples, various devices utilized, and different cut-off points. Another limitation was the small variety of natural disasters studied, since most of the previous studies were conducted after the earthquakes and tsunamis. Thus, it is recommended to study the prevalence rate of PGD in the survivors of other natural disasters such as floods and fires, as well as unnatural disasters, such as wars, due to the difference in the nature of physical and psychological injuries happened by each. The results of this study showed that the prevalence of PGD among the survivors of natural disasters was 38.81 %, and with the passage of time, the trend rate decreased.

The nature of the death of loved ones after natural disasters has many of the risk factors affecting the prevalence of PGD among the survivors. What adversely affect the prevalence of PGD after natural disasters are the disruption and slowness of actions required for the survivors in the recovery phase and the prolongation of the process of returning to normal life.

Dispatching specialized teams trained in psycho-spiritual counseling, rapid screening, and providing rehabilitation services in all four dimensions of survivors' health after natural disasters are among factors that can effectively prevent PGD. The policy-making and planning of the organizations responsible for disaster management are suggested to be prepared for the quick settlement of the survivors and reconstruction of the damaged buildings.

## Ethics approval and consent to participate

This study was approved by the Research Committee of 10.13039/501100013778Aja University of Medical Sciences, Tehran, Iran (ethical code: IR.AJAUMS.REC.1402.203).

## Funding

The study was supported by 10.13039/501100013778Aja University of Medical Sciences, Tehran, Iran.

## Competing interests

The authors declared no competing interests.

## Implications for policy and Practice

•Training spiritual-psychological counselors and planning their timely dispatch to the disaster area.
•Providing spiritual rehabilitation programs for the bereaved and clinical pastoral care for those injured and hospitalized
•Planning for the rescue, transfer and treatment of the injured immediately.

## Declaration of competing interest

The authors declare that they have no known competing financial interests or personal relationships that could have appeared to influence the work reported in this paper.
